# Comment on the "Mortality Rate and Years of Life Lost Due to Burns in Southern Iran during 2004–2019: A Population-Based Study"

**DOI:** 10.34172/aim.2024.08

**Published:** 2024-01-01

**Authors:** Mehran Rostami, Mohammad Jalilian

**Affiliations:** ^1^Deputy of Health, Kermanshah University of Medical Sciences, Kermanshah, Iran; ^2^Department of Clinical Sciences, Faculty of Veterinary Medicine, Islamic Azad University, Garmsar Branch, Semnan, Iran

 We want to comment on the paper authored by Mirahmadizadeh and colleagues entitled “Mortality rate and years of life lost due to burns in Southern Iran during 2004–2019: A population-based study” recently published in *Archives of Iranian Medicine* in 2023.^1^

 For a more precise interpretation of the results obtained for this study, it is recommended that several essential points are considered collectively. After reviewing these aspects anew, it is feasible to draw additional conclusions. Initially, referring to the research conducted by Kazemzadeh et al, it is emphasized that burns have been a significant health concern for children in Iran. After accidents, burns constitute the second most common cause of death in this age group. Notably, the study focused on children under the age of five.^2^ Throughout the study of Mirahmadizadeh et al, around 39% of burn-related deaths occurred within the 15 to 29 age group. This specific demographic group is notably regarded as the most susceptible to mortality resulting from burns. In the mentioned study, visual representations in Figures 1 and 2 validate the notable relationship between the number of deaths and the corresponding years of life lost, emphasizing significantly lower values within the under-five age group compared to the 15 to 29 age group. Although this initial observation seems logical due to the broader age range of the latter group, a more detailed analysis of the constituents of this scenario is necessary for a comprehensive understanding.

 For example, research has previously identified individuals under 30 as the main group affected by complete suicide in Iran.^3^ Interestingly, the results of the mentioned study are closely similar to the findings of these earlier investigations. However, Mirahmadizadeh et al specify the self-immolation cases as exclusion criteria. This exclusion prompts an inquiry into how the true nature of this finding can be ascertained? To address this question, several crucial factors need to be considered. By assembling these elements, a more enhanced understanding of this puzzling scenario can be achieved. It is essential to highlight that the data collection process should also be acknowledged as a vital aspect in this context.

 One data collection method includes a verbal autopsy, which entails interviewing family members as well as others regarding deaths that occurred outside hospitals, health centers, remote rural areas, and forensic medical centers.^4,5^ In Iran, a Middle Eastern country where majority of the people are Muslims, suicide is a subject with social stigma. Therefore, some families are unwilling to disclose complete suicide as a cause of death. In some cases, the stigma might lead individuals to attribute the deceased’s intention to accidental rather than deliberate actions.^6^ This skewed tendency could be particularly pronounced burn-related fatality in some cases. Consequently, the misclassification of burn deaths might not be accurately recorded in the self-immolation databases. This could lead to an underestimation of complete suicide in the country.^6^ Moreover, due to misclassification of the cause of death, burn-related fatalities might be analyzed collectively, including intentional and unintentional deaths. This could lead to an overestimation of burn-related deaths in the study.

 Let’s look at the results of Mirahmadizadeh et al from this perspective. Hence, acknowledging this point as a significant limitation that could affect the study results is essential. Moreover, it is crucial to mention that the data collection process should also be considered from a holistic perspective.
